# Harnessing large language models in virtual CBT for university students’ academic anxiety: a preliminary randomized trial

**DOI:** 10.3389/fpubh.2026.1874822

**Published:** 2026-08-27

**Authors:** Hao Fang, Zixi Huang, Lingxin Zhu, Qinling Dai, Minjian Hong, Hongyun Guo, Encong Wang

**Affiliations:** 1 Wuhan Institute of Technology, Wuhan, China; 2 China University of Geosciences, Wuhan, China; 3 Southwest Forestry University, Kunming, China; 4 Beijing Chaoyang Hospital Affiliated to Capital Medical University, Beijing, China

**Keywords:** academic anxiety, cognitive behavioral therapy, large language model, virtual agent, virtual reality

## Abstract

Academic anxiety is a common mental health problem among university students. It is often associated with reduced learning efficiency and an increased risk of depression. Cognitive behavioral therapy (CBT) is supported by an established evidence base, but its use in university settings is still limited by factors such as therapist availability, time, and physical space. To explore a new approach to digitally assisted intervention, this study integrated virtual reality (VR), large language model (LLM), and retrieval-augmented generation (RAG) technologies. We developed an LLM-VR-CBT system based on CBT principles and preliminarily examined its short-term intervention effects on academic anxiety among university students. This study used a three-arm randomized controlled design. A total of 60 university students with academic anxiety were included and randomly assigned to the LLM-VR-CBT group, traditional CBT group, or minimal-support control group, with 20 participants in each group. The intervention lasted 4 weeks. The linear mixed-effects model results showed significant group-by-time interactions for academic anxiety and heart rate. The LLM-VR-CBT group and traditional CBT group showed significantly greater reductions in academic anxiety and heart rate than the minimal-support control group. The difference in change between the two active intervention groups did not reach statistical significance. Skin temperature did not show a significant group-by-time interaction. These findings suggest that the LLM-VR-CBT system may help reduce academic anxiety among university students in the short term. Because this was a small, short-term exploratory trial and adverse events were not systematically collected as prespecified safety endpoints, future studies with larger samples, multicenter designs, long-term follow-up, and predefined safety monitoring are needed to further evaluate the system’s stability, safety, feasibility, and application boundaries.

## 1 Introduction

Academic anxiety among university students is a common psychological difficulty in higher education settings. It is mainly characterized by excessive worry, tension, negative cognition, and avoidance behaviors in learning, examination, and academic evaluation contexts. It may also be associated with poorer academic performance, sleep problems, impaired social functioning, and an increased risk of depression (1–3). From a psychological mechanism perspective, catastrophic thinking, self-negation, maladaptive emotion regulation, and low academic self-efficacy are considered closely related to academic anxiety (4–6). CBT has been widely used for anxiety-related problems and academic stress interventions. It helps individuals identify and adjust irrational beliefs through cognitive restructuring, exposure exercises, behavioral activation, and problem-solving training, thereby reducing avoidance and procrastination behaviors (7, 8). However, in university mental health services, traditional face-to-face CBT is still limited by therapist availability, time, space, and mental health stigma. Students may still find it difficult to seek help actively and remain engaged (9).

Digital CBT, VR, and virtual agents provide new possibilities for improving intervention accessibility and contextual adaptability. Existing studies suggest that internet-based CBT and digital CBT can provide some support for anxiety-related problems among university students (10–12). VR can support exposure training through controllable, repeatable, and presence-enhancing virtual environments. It has been used in contexts such as specific phobias, social anxiety, public speaking anxiety, and exam-related anxiety (13–16). At the same time, virtual agents and embodied conversational agents can enhance the sense of interaction and engagement through speech, facial expressions, and nonverbal feedback (17–20). However, existing VR-CBT or virtual agent interventions often rely on predefined scripts and fixed task procedures. They are often difficult to adapt flexibly to students’ real-time expressions and specific academic stress themes. They also have difficulty covering diverse academic stress contexts such as examinations, thesis writing, classroom presentations, procrastination, and self-negation at the same time.

Large language models (LLMs) and RAG technologies provide a new technical pathway for addressing these issues. LLMs show application potential in natural language understanding, emotional support, and personalized responses. RAG can ground model-generated content in an external knowledge base and improve the relevance between responses, professional knowledge, and specific contexts (17, 18, 21–23). In academic anxiety interventions, an LLM-RAG module may identify specific stress themes and negative automatic thoughts based on students’ language input. Under the constraints of predefined CBT procedures, a professional knowledge base, and safety rules, it may provide more contextualized cognitive restructuring, emotion regulation, and behavioral recommendations. However, studies that integrate an LLM-RAG-supported virtual agent, VR-based academic stress contexts, and CBT procedures, and empirically examine this integration through a randomized controlled design, remain limited.

This study developed an LLM-VR-CBT system that integrates an LLM-RAG-supported virtual agent with VR-based academic stress contexts. Within a CBT framework, we preliminarily examined its short-term intervention effects on academic anxiety among university students. This study used a three-arm randomized controlled trial design to compare pre-post changes in academic anxiety and related physiological indicators under the LLM-VR-CBT intervention, traditional face-to-face CBT intervention, and minimal-support control condition. The focus of this study was to examine differences in changes over time across groups and to explore whether improvement in subjective anxiety was accompanied by changes in physiological indicators such as heart rate and skin temperature. Given the limitations of sample size and study duration, this study is positioned as a preliminary exploratory study. It is not intended as a definitive validation of the system’s clinical efficacy, equivalence, no inferiority, or large-scale implementation value.

## 2 Methods

### 2.1 Study design

This study used a three-arm parallel randomized controlled trial (RCT) design to compare the effects of the LLM-VR-CBT system intervention, traditional face-to-face CBT intervention, and minimal-support control condition in reducing academic anxiety among university students. The study used a 3 (intervention condition: LLM-VR-CBT group, traditional CBT group, and minimal-support control group) × 2 (time point: pre-test and post-test) mixed design. Because the intervention conditions differed clearly in their implementation formats, participants could not be fully blinded. However, all outcome assessors and statisticians remained blinded to group allocation to reduce observer bias during assessment and analysis. Randomization was conducted using stratified randomization by sex and anxiety severity, including mild anxiety (AAS score 15–25) and moderate anxiety (AAS score 26–44). A 1:1:1 allocation ratio was generated using the random number generator in Excel, and allocation concealment was implemented independently by a third party. The study lasted 6 weeks, including a pre-test phase (T0, baseline measurement), an intervention phase (T1–T4, once per week, 50 min per session), and a post-test phase (T5).

This study was reviewed and approved by the Ethics Committee of Wuhan Institute of Technology. This study was a single-center, small-sample preliminary exploratory RCT and was not prospectively registered on a public clinical trial registry.

### 2.2 Participants

Participants were undergraduate and graduate students from a university in Wuhan. They were recruited through referrals from the campus mental health center, campus public account announcements, and class presentations. Because recruitment mainly used open campus-based announcements, referrals, and class presentations, the research team did not systematically record the number of all students who were initially exposed to or viewed the recruitment information. Thus, the participant flow diagram begins with students who actively contacted the research team and completed formal eligibility assessment. All participants completed eligibility screening, including the Academic Anxiety Scale (AAS), an in-person interview, and a VR adaptability test. The inclusion criteria were as follows: age 18–26 years; AAS total score of at least 15; no psychotherapy or medication adjustment during the past 3 months; no contraindications to VR use; and ability to sign informed consent and commit to completing all study procedures. The exclusion criteria included meeting DSM-5 diagnostic criteria for major mental disorders (24), clear self-harm or suicide risk, previous CBT or VR intervention experience, severe physical disease, or inability to complete all measurements because of practical reasons.

Based on the moderate-to-large intervention effects reported in previous studies on CBT, digital CBT, and test/academic anxiety interventions, this study used Cohen’s *d* = 0.65 as the planning effect size for exploratory sample size estimation (7, 11, 12, 17, 18). It should be noted that existing related studies more often report Cohen’s *d* for between-group differences or within-group pre-post changes, and less often directly report Cohen’s *f* for the group-by-time interaction in a 3 × 2 mixed design. Thus, this study did not treat *d* = 0.65 as a precise prior estimate of the interaction effect. Instead, it used this value as an approximate planning value for a preliminary exploratory study.

This study used G*Power 3.1 for *a priori* sample size estimation (25). Given the 3 (group: LLM-VR-CBT group, traditional CBT group, and minimal-support control group) × 2 (time: pre-test and post-test) mixed experimental design, the sample size estimation used repeated measures ANOVA, within–between interaction under *F* tests as an approximate calculation model. The group-by-time interaction was specified as the main effect of interest. Because G*Power requires Cohen’s *f* as input in this model, this study followed Cohen’s approximate relationship, *d* ≈ 2*f*, and converted the planned effect size of Cohen’s *d* = 0.65 to Cohen’s *f* = 0.325 (26). This conversion was used only for sample size planning and did not mean that this study prespecified a precise interaction effect size.

With a significance level of *α* = 0.05, statistical power of 1 − *β* = 0.80, number of groups = 3, number of measurements = 2, repeated-measures correlation = 0.50, and nonsphericity correction *ε* = 1, the estimated sample size was approximately 20 participants per group. Thus, this study planned to include a total sample of 60 participants, with 20 participants in each group. It should be emphasized that this sample size estimation was mainly intended to support this study as a single-center, small-sample preliminary exploratory RCT for the initial examination of short-term intervention effects. It was not intended to provide sufficient statistical power for definitive comparisons between the active intervention groups, nor was it used for formal equivalence or no inferiority testing.

**Figure 1:**
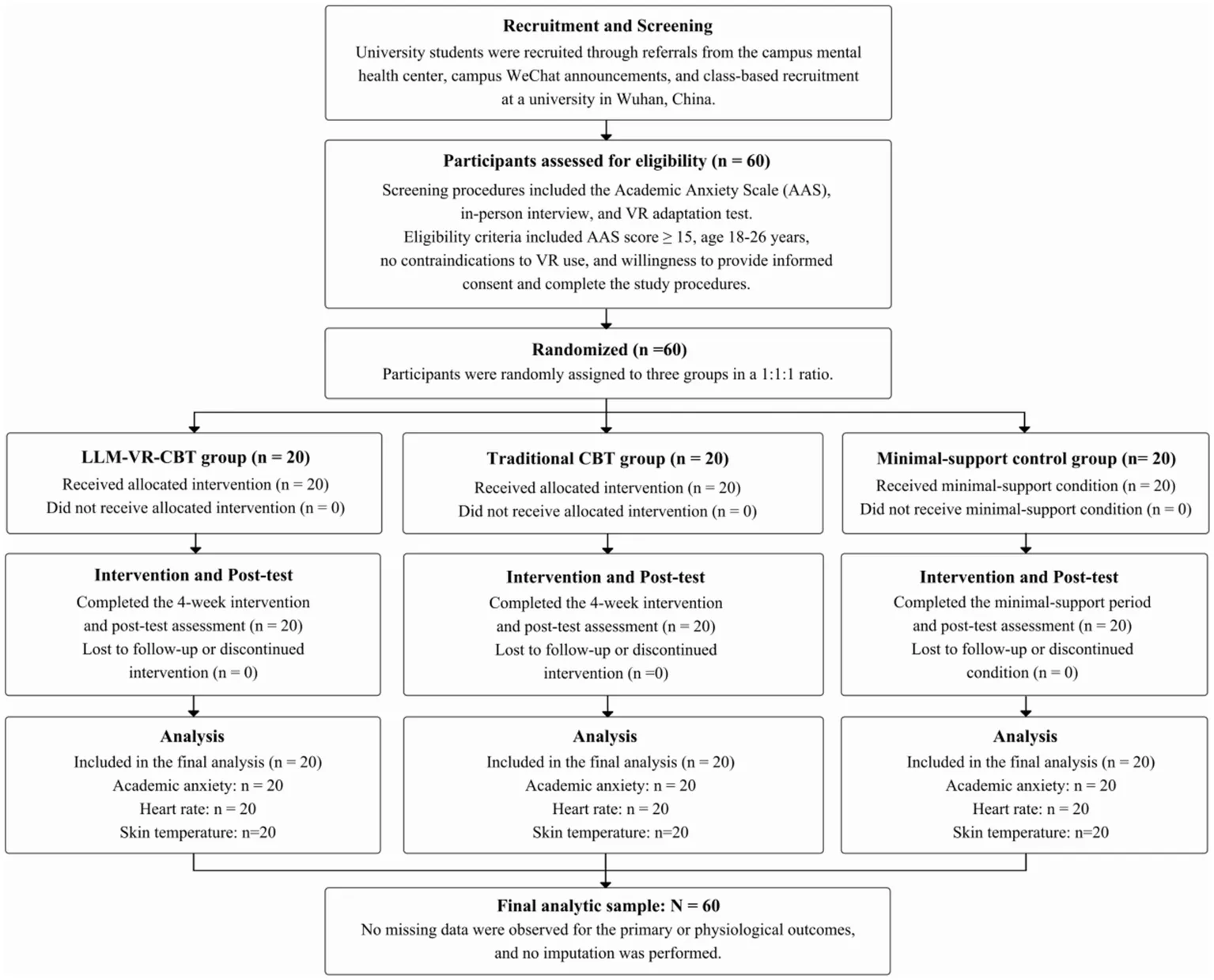
Participant flow diagram following CONSORT guidelines. Because the early recruitment stage of this study used open campus-based announcements and class presentations, the number of students who were initially exposed to or viewed the recruitment information was not systematically recorded. Thus, the flow diagram begins with students who actively contacted the research team and completed formal eligibility assessment.

A total of 60 participants who met the inclusion criteria signed informed consent and were randomly assigned to the LLM-VR-CBT group, traditional CBT group, and minimal-support control group, with 20 participants in each group. All participants completed the pre-test, the corresponding intervention or minimal-support control condition, and the post-test assessment, and were included in the final analysis. Baseline characteristics of the three groups are shown in Supplementary Table S1, including age, sex, academic level, major type, AAS severity, AAS total score, mean heart rate, and mean skin temperature. Overall, the three groups showed similar distributions in demographic characteristics, AAS severity, main baseline outcomes, and physiological indicators, with no clear baseline imbalance. All participants completed the pre-test and post-test assessments. There were no missing data for the primary outcome, the AAS, or the physiological indicators, heart rate and skin temperature. Thus, this study used complete data for analysis, and no missing-value imputation was performed Figure 1.

### 2.3 Intervention

**Figure 2:**
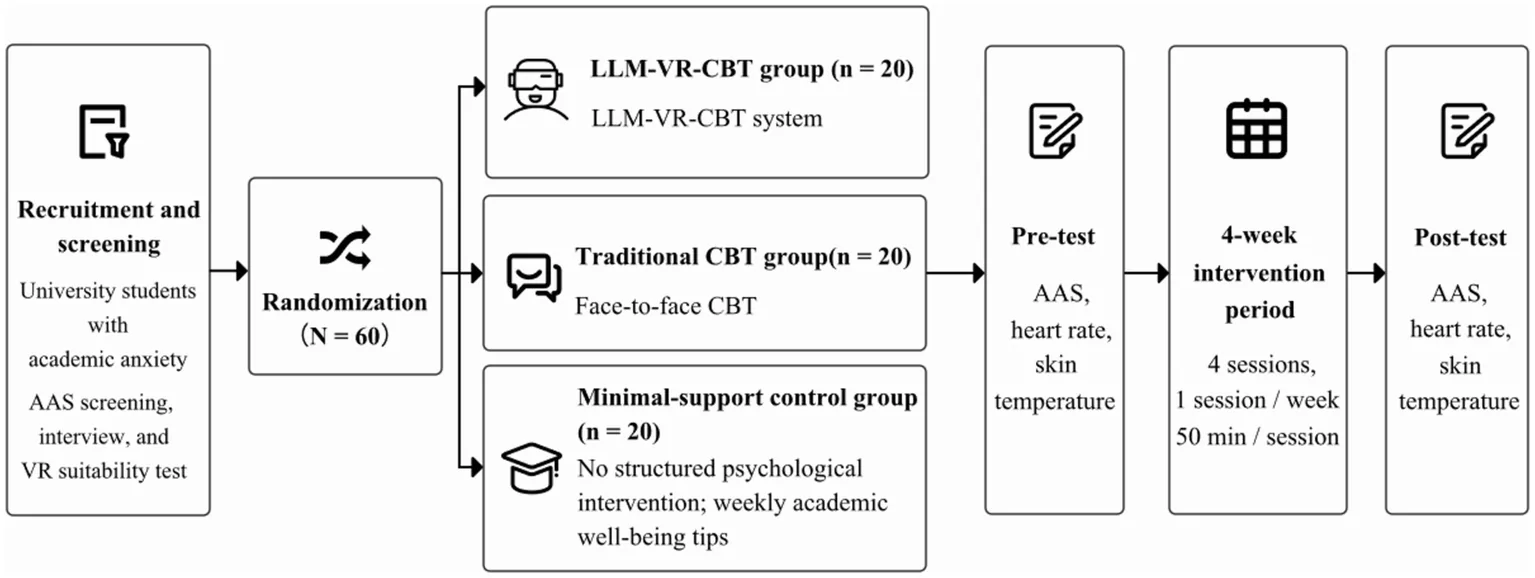
Overall study design.

This study included three experimental conditions: the LLM-VR-CBT group, the traditional CBT group, and the minimal-support control group. The LLM-VR-CBT group received the intervention provided by the LLM-VR-CBT system. The traditional CBT group received face-to-face CBT. The minimal-support control group did not receive a structured psychological intervention. Participants in this group maintained their usual study and daily life and received one “academic well-being tip” each week during the intervention period. All three groups completed the same pre-test and post-test assessment procedures. The overall study design is shown in Figure 2.

The system design of the LLM-VR-CBT group was based on CBT theory (27, 28). It focused on negative automatic thoughts, avoidance behaviors, emotional arousal, and task procrastination in academic anxiety, and included four core modules: cognitive restructuring, virtual exposure, behavioral activation, and emotion regulation. In the cognitive restructuring module, the system used Socratic questioning to guide participants to identify catastrophic thoughts and irrational beliefs related to contexts such as examinations, classroom presentations, and thesis writing. It also helped them form more adaptive alternative thoughts (27). In the virtual exposure module, the system simulated typical academic stress contexts through VR scenarios, allowing participants to complete gradual exposure exercises in a relatively controlled virtual environment (15, 29, 30). In the behavioral activation module, the system helped participants break down academic tasks, develop study plans, and reduce procrastination behaviors (31). In the emotion regulation module, the system provided breathing training, mindfulness exercises, and emotional stabilization strategies according to participants’ current status, in order to reduce anxiety-related physiological arousal (32, 33).

The virtual agent module in the LLM-VR-CBT system used ERNIE Bot 4.0 as the base model (34), and combined RAG technology to construct a knowledge system for academic anxiety intervention (23). The system did not use the LLM as an independent therapeutic agent. Instead, under predefined CBT intervention procedures, RAG knowledge-base constraints, and human supervision mechanisms, it assisted with cognitive guidance, emotional support, behavioral recommendations, and exposure training feedback (35). The system knowledge base mainly consisted of three types of materials. The first type included theories, scales, and intervention studies related to academic anxiety. The second type included professional materials on CBT counseling techniques, cognitive restructuring, behavioral activation, exposure training, and emotion regulation. The third type included dialogue materials developed based on expert interviews and common academic stress contexts. Materials included in the knowledge base had to meet the following criteria: clear source, direct relevance to academic anxiety or CBT intervention, language suitable for university students, and no unverified high-risk clinical advice.

In terms of the interaction workflow, the system followed the basic pathway of “user input identification—knowledge-base retrieval—prompt template constraint—LLM generation—safety rule review—feedback output.” When participants entered emotions and thoughts related to academic stress, the system first identified the main problem type. It then retrieved relevant CBT techniques and contextualized coping strategies from the knowledge base and used the retrieved content as contextual input for the prompt template. This constrained the response scope of the model and reduced the risk of hallucination and deviation from CBT principles. The virtual agent then interacted with participants through speech, facial expressions, and text feedback. Existing studies show that health care chatbots and embodied conversational agents can support mental health interventions and user engagement through natural language interaction, nonverbal cues, and continuous feedback (20, 36, 37).

**Figure 3:**
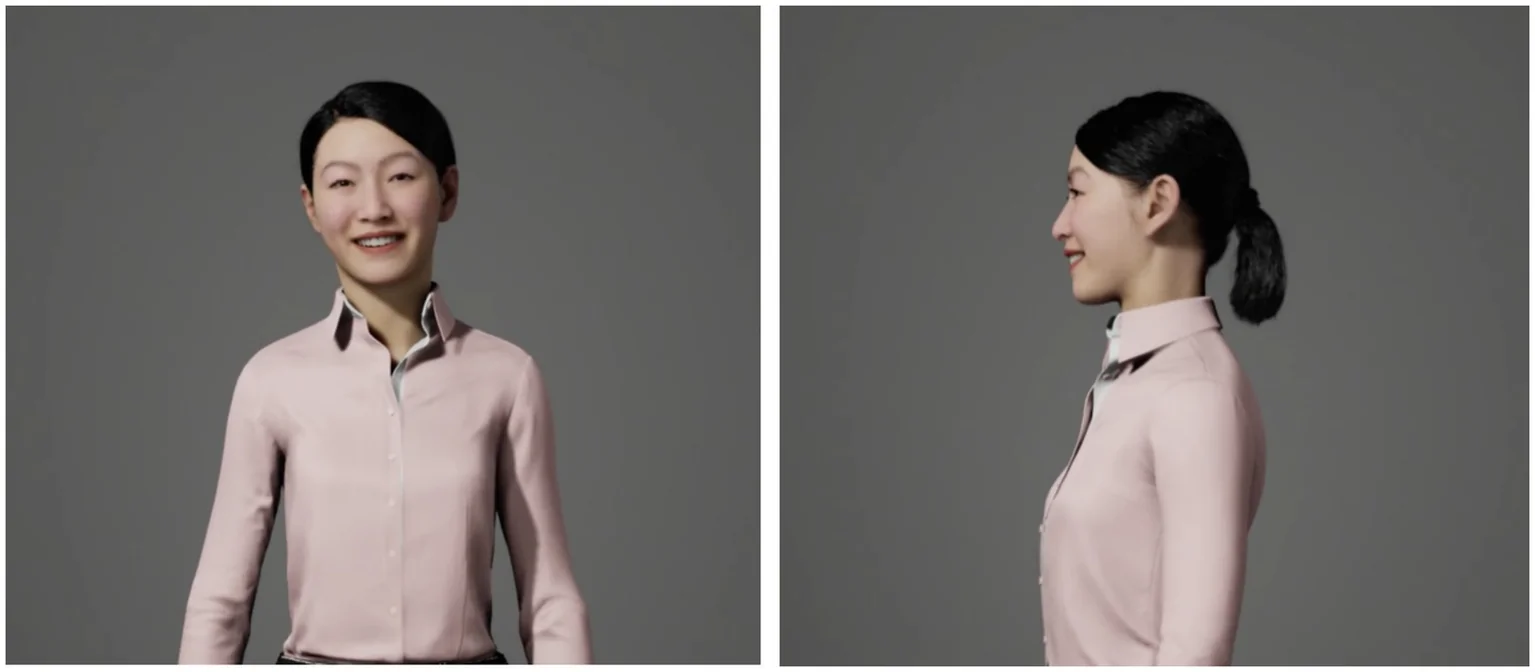
Virtual agent avatar used in the LLM-VR-CBT intervention.

The virtual human avatar (Figure 3) was generated using MetaHuman Creator and imported into the Unreal Engine 5 scene through Quixel Bridge. The virtual agent was designed as a gentle and professional psychological instructor, providing speech, text, and nonverbal feedback during the LLM-VR-CBT intervention. The system enhanced the sense of embodied interaction through nonverbal behaviors such as facial expressions, head orientation, nodding feedback, moderate gestures, and posture changes. Speech interaction followed the workflow of “user speech input—speech-to-text—LLM text generation—text-to-speech output,” allowing participants to interact with the virtual agent in a relatively natural conversational manner.

**Figure 4:**
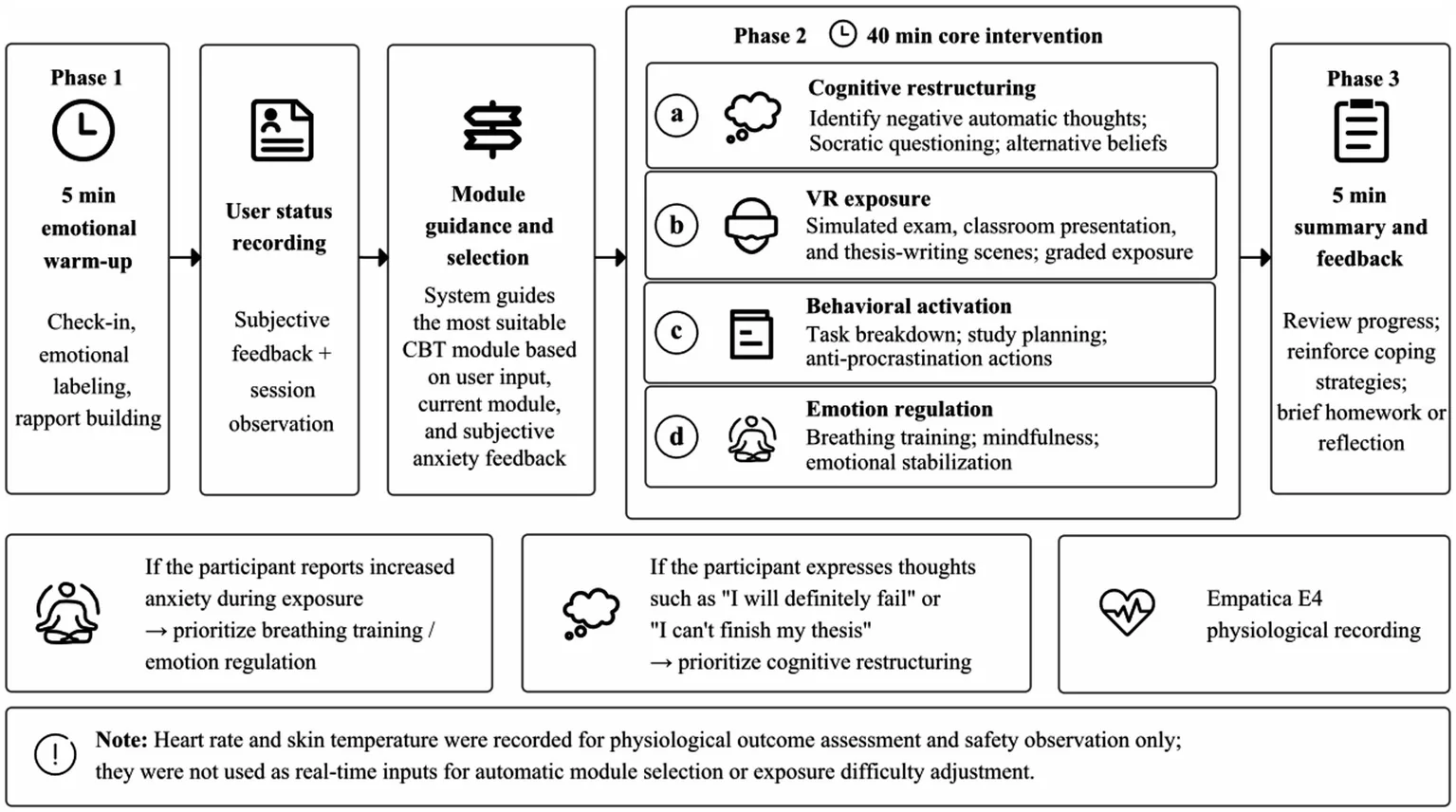
Procedure of each LLM-VR-CBT session.

Each LLM-VR-CBT intervention session lasted 50 min, including a 5-min emotional warm-up, a 40-min core intervention, and a 5-min summary and feedback phase. During the core intervention phase, the system guided participants among the cognitive restructuring, virtual exposure, behavioral activation, and emotion regulation modules according to their language input, current intervention module, and subjective anxiety feedback. For example, when a participant showed clear anxiety responses in an exposure scenario, the system could prioritize the breathing training or emotion regulation module. When a participant expressed negative automatic thoughts such as “I will definitely fail” or “I cannot finish my thesis,” the system prioritized the cognitive restructuring module for guidance. The procedure of each LLM-VR-CBT session is shown in Figure 4.

To reduce the risk that the LLM might generate inappropriate psychological advice, the system included multilayered safety rules. First, model outputs were limited to academic anxiety support, CBT technique guidance, and general psychoeducation, and did not provide psychiatric diagnosis, medication advice, or crisis intervention conclusions. Second, when participants expressed self-harm, suicidal ideation, severe depression, psychotic symptoms, or other high-risk content, the system did not continue automated intervention. Instead, it output a crisis prompt and recommended that participants immediately contact the campus mental health center or professional staff. Third, all intervention dialogues and key interaction records were retained for later quality control and human review. Fourth, system outputs were required to follow the principles of supportiveness, nonjudgment, evidence-based guidance, and non-replacement of professional psychological counselors.

To reduce the risk of potentially inappropriate responses and insufficient handling of psychological risks during the LLM-VR-CBT intervention, the research team established safety rules, system boundaries, and human supervision procedures. During the LLM-VR-CBT intervention, researchers with mental health service experience provided on-site or back-end supervision. They could pause the intervention and provide human intervention in a timely manner when participants showed clear discomfort, when system responses clearly deviated from intervention goals, or when high-risk psychological expressions appeared. Two independent experts with CBT experience conducted quality reviews of randomly selected intervention audio recordings and dialogue logs. They assessed whether system responses were consistent with CBT intervention principles, whether they deviated from intervention goals, and whether there were potentially inappropriate responses.

It should be noted that safety and intervention fidelity records in this study were mainly used for trial process monitoring and *post hoc* description. They were not prespecified independent safety endpoints. The confirmable records during the study mainly included the number of planned sessions, number of completed sessions, session completion rate, human supervision method, and expert review method. VR-related physical discomfort, physical or psychological adverse events, risk alerts for high-risk psychological content, the number of responses blocked or modified by safety rules, technical failures, human correction of agent responses, referrals to professional psychological support, and protocol deviations were not systematically collected as prespecified quantitative indicators. This study therefore summarizes the available process information in Supplementary Table S2 and explicitly states the limited nature of safety evidence in the limitations section. To further improve the descriptive transparency and process auditability of the technical intervention, this article added an LLM-RAG technical transparency table in the Appendix. This table reports the base model and calling platform, RAG architecture, knowledge-base sources, corpus screening and annotation criteria, platform default parameters, prompt template constraints, hallucination control strategies, human supervision workflow, psychological risk escalation procedures, and example interaction content.

The traditional CBT group received four face-to-face sessions delivered by experienced licensed psychological counselors according to a standardized intervention manual. Each session lasted 50 min. The content included therapeutic alliance building, identification of negative automatic thoughts, cognitive restructuring, behavioral exposure, problem-solving training, and relapse prevention. The minimal-support control group did not receive a structured psychological intervention. Participants in this group only received one “academic well-being tip” each week and maintained their usual study and daily life. After the study ended, participants in this group were allowed to experience the LLM-VR-CBT system.

### 2.4 Measures

This study used the AAS as the primary outcome measure to assess overall anxiety level. Heart rate and skin temperature were selected as objective supplementary physiological indicators and were collected using the Empatica E4 wristband to explore whether changes in academic anxiety were accompanied by changes in physiological arousal. Heart rate was mainly used to reflect anxiety-related physiological arousal. Skin temperature was reported as an exploratory peripheral physiological indicator to assist in observing possible physiological changes before and after the intervention. Because skin temperature can be affected by factors such as ambient temperature, wearing position, and peripheral vascular responses, this study interpreted this indicator cautiously.

It should be noted that the prespecified outcome measures of this RCT were AAS score, mean heart rate, and mean skin temperature. Process or experiential indicators, including presence, usability, satisfaction, engagement, interaction quality, module fidelity, and immersive experience, were not systematically collected or statistically analyzed as formal outcome measures in this RCT. Thus, the statements in this article about personalized interaction, contextualized support, and immersive experience are mainly used to describe the system design intention and possible pathways of action, rather than to indicate that these mechanisms were directly verified in this trial.

### 2.5 Academic anxiety scale

Academic anxiety was measured using the Academic Anxiety Scale (AAS) developed by Cassady et al. (38). The scale is used to assess learners’ perceived academic stress and anxiety responses in educational settings. The items used in this study were based on publicly available AAS materials and related original literature, and were linguistically and contextually adapted according to Chinese university students’ learning, examination, classroom task, and academic evaluation contexts.

Specifically, the research team first translated the original English items into Chinese. While preserving the meanings of the original items, we adjusted some wording in the Chinese context to better fit how Chinese undergraduate and graduate students understand academic stress contexts. The research team then reviewed the semantic clarity, contextual applicability, and potential risk of misunderstanding of the Chinese items based on previous user interviews and expert interviews. The previous expert interviews involved three campus psychological counselors with experience in university mental health counseling and CBT intervention. The interviews covered the main manifestations of academic anxiety, use of assessment tools, key points of CBT intervention, and design requirements for the virtual system. Based on expert feedback, the research team refined some Chinese wording but did not change the number of items, scoring method, or total score calculation. This study did not conduct a separate large-sample psychometric validation of the Chinese AAS version. Thus, this version is more accurately described as a language and context-adapted version based on the original AAS, rather than a revalidated Chinese revised scale.

The AAS version actually administered in this study included 11 items and used a 4-point Likert scale, ranging from 1 (“completely inconsistent”) to 4 (“completely consistent”). Scores for all items were directly summed to produce a total score, with a total score range of 11–44. Higher scores indicate higher levels of academic anxiety. According to the AAS severity classification proposed by Finch et al. (39), this study used a total score ≥15 as the screening criterion for evident academic anxiety. Scores of 15–25 indicate mild anxiety, and scores of 26–44 indicate moderate anxiety. It should be noted that this cutoff was used for sample screening and severity stratification in this study, rather than as a clinical diagnostic criterion. Participant inclusion was also determined in combination with an in-person interview and a VR adaptability test. In the formal data analysis, eligibility screening, anxiety severity stratification, stratified randomization, and the primary outcome analysis were all based on the total score of this 11-item version. In this study sample, the scale showed good internal consistency, with Cronbach’s *α* = 0.90.

### 2.6 Physiological indicators

Physiological indicators were collected using the Empatica E4 wristband. This device records heart rate through photoplethysmography signals and records skin temperature through a temperature sensor. This study used heart rate as the main supplementary outcome indicator reflecting changes in physiological arousal and reported skin temperature as an exploratory peripheral physiological indicator. It should be noted that heart rate and skin temperature were used only to assess pre-post physiological changes. They were not used as real-time physiological inputs to the LLM-VR-CBT system, nor were they used to automatically trigger module switching, adjust exposure task difficulty, or change virtual scene content. Before data collection, participants were asked to sit quietly for 10 min in a temperature-controlled (22–25 °C) quiet environment to stabilize their physiological state and reduce the influence of environmental factors and short-term activity on physiological indicators. The heart rate sensor was worn on the participant’s nondominant wrist, and the skin temperature sensor was attached to the palmar side of the index finger to ensure stable signal acquisition. The formal statistical analysis used mean heart rate and mean skin temperature values before and after the intervention. Raw data were preprocessed using Empatica E4 Manager software (Version 1.10.0). After clear outliers were removed, the mean value of valid data was calculated as the final statistical indicator. If the device was worn for continuous recording during the intervention, its role was limited to research recording and safety observation, and it did not enter the system decision-making process.

### 2.7 Statistical analyses

This study used RStudio for statistical analyses. Data organization and result export were performed using the readxl, dplyr, tidyr, and writexl packages. LMMs were constructed using the lmer() function in the lme4 package. Fixed-effect *F* tests, Satterthwaite approximate degrees of freedom, and *p* values were obtained using the lmerTest package. Estimated marginal means and *post hoc* comparisons were obtained using the emmeans package. Partial *η*^2^ was converted from *F* values and degrees of freedom using the effectsize package. Basic model diagnostics were conducted using the performance package.

To align the statistical method with the 3 (intervention condition: LLM-VR-CBT group, traditional CBT group, and minimal-support control group) × 2 (time point: pre-test and post-test) mixed experimental design, this study built separate LMMs with academic anxiety score, mean heart rate, and mean skin temperature as dependent variables. In the models, group, time, and the group-by-time interaction were specified as fixed effects, and participant ID was specified as a random intercept to control for correlations between repeated measurements from the same participant. The models were fitted using maximum likelihood estimation (REML = FALSE). Group and time were both treated as categorical variables. Group included the LLM-VR-CBT group, traditional CBT group, and minimal-support control group, and time included pre-test and post-test. For Type III fixed-effect tests, categorical variables were coded using sum-to-zero contrasts.

The model form was as follows:

*Y_*ij*_* = *β₀* + *β₁Group_*i*_* + *β₂Time_*j*_* + *β₃*(*Group_*i*_* × *Time_*j*_*) + *u_*i*_* + *ε_*ij*_.*

where *Y_*ij*_* denotes the outcome for participant *i* at time point *j*; *Group_*i*_* denotes the group to which the participant belonged; *Time*j** denotes the measurement time point; *Group_*i*_* × *Time_*j*_* denotes the group-by-time interaction; *u_*i*_* denotes the participant-level random intercept; and *ε_*ij*_* denotes the residual error. The group-by-time interaction was the primary test term in this study and was used to determine whether the magnitude of change from pre-test to post-test differed significantly across intervention conditions.

For fixed-effect results, this study reports *F* values, degrees of freedom, *p* values, and partial *η*^2^. In the LMM context, partial *η*^2^ was converted from the Type III *F* test results using the following formula: partial *η*^2^ = (*F* × *df*effect)/(*F* × *df*effect + *df*error). This effect size describes the relative proportion of variance explained by each fixed effect after controlling for other model terms.

When the group-by-time interaction was significant, this study further used estimated marginal means for *post hoc* comparisons. Within-group change was defined as Post—Pre. Between-group change-score comparison was defined as the difference in pre-post change between groups, for example, (Post—Pre)LLM-VR-CBT − (Post—Pre)minimal-support control. For academic anxiety and heart rate, a negative value indicated that the former group showed a greater decrease than the latter group. The Holm method was used for multiple-comparison correction. This correction was applied separately within post hoc comparisons for each outcome indicator, rather than across all outcome indicators, because academic anxiety was the primary outcome, whereas heart rate and skin temperature were supplementary or exploratory physiological indicators.

Before interpreting the models, this study conducted basic diagnostics for each LMM, including checks of residual normality, heteroscedasticity, model singularity, and outliers. Model diagnostics were used to assess whether the basic assumptions of the LMM were clearly violated and to support the robustness of result interpretation.

## 3 Results

### 3.1 Descriptive statistics

Table 1 shows the pre-test and post-test descriptive statistics for academic anxiety score, mean heart rate, and mean skin temperature in the three groups. The results showed that academic anxiety scores decreased after the intervention in both the LLM-VR-CBT group and the traditional CBT group, whereas the minimal-support control group showed only small pre-post changes. Mean heart rate showed a similar trend. Heart rate decreased after the intervention in the LLM-VR-CBT group and the traditional CBT group, whereas the minimal-support control group did not show a decreasing trend. Mean skin temperature changed only slightly in all three groups.

**Table 1:** Descriptive statistics for outcome measures at pre-test and post-test by group.

Outcome	Group	*n*	Pre-test M *(*SD*)*	Post-test M *(*SD*)*
Academic anxiety	LLM-VR-CBT group	20	31.15 (5.64)	27.15 (4.45)
Academic anxiety	Traditional CBT group	20	31.50 (5.82)	25.45 (4.12)
Academic anxiety	Minimal-support control group	20	31.80 (4.89)	31.35 (3.94)
Mean heart rate	LLM-VR-CBT group	20	80.05 (4.82)	75.10 (4.87)
Mean heart rate	Traditional CBT group	20	80.50 (4.49)	74.35 (4.80)
Mean heart rate	Minimal-support control group	20	80.05 (5.11)	81.25 (4.60)
Mean skin temperature	LLM-VR-CBT group	20	30.18 (0.57)	30.26 (0.44)
Mean skin temperature	Traditional CBT group	20	30.11 (0.34)	30.14 (0.39)
Mean skin temperature	Minimal-support control group	20	30.04 (0.52)	30.06 (0.51)

### 3.2 Linear mixed-effects model analysis results

#### 3.2.1 Academic anxiety score

A linear mixed-effects model (LMM) was established with academic anxiety score as the dependent variable. The results are shown in Table 2. Table 2 shows that the main effect of time was significant, *F*(1, 60) = 50.348, *p* < 0.001, indicating that participants’ academic anxiety levels changed significantly from pre-test to post-test overall. The group-by-time interaction was also significant, *F*(2, 60) = 10.998, *p* < 0.001, partial *η^2^* = 0.268, indicating that the magnitude of pre-post change in academic anxiety differed significantly across the three groups. This result indicates that changes in academic anxiety differed across intervention conditions.

**Table 2:** Linear mixed-effects model analysis results for outcome measures.

Outcome	Fixed effect	*F*(*df*1, *df*2)	*p*	partial *η*^2^
Academic anxiety	Group	*F*(2, 60) = 2.824	0.067	0.086
Academic anxiety	Time	*F*(1, 60) = 50.348	<0.001	0.456
Academic anxiety	Group × time	*F*(2, 60) = 10.998	<0.001	0.268
Mean heart rate	Group	*F*(2, 60) = 3.525	0.036	0.105
Mean heart rate	Time	*F*(1, 60) = 55.163	<0.001	0.479
Mean heart rate	Group × time	*F*(2, 60) = 26.252	<0.001	0.467
Mean skin temperature	Group	*F*(2, 60) = 0.778	0.464	0.025
Mean skin temperature	Time	*F*(1, 60) = 1.476	0.229	0.024
Mean skin temperature	Group × time	*F*(2, 60) = 0.291	0.748	0.010

The group-by-time interaction was the primary test term in this study and was used to determine whether the magnitude of change from pre-test to post-test differed significantly across groups.

#### 3.2.2 Mean heart rate

A linear mixed-effects model was established with mean heart rate as the dependent variable. The results are shown in Table 2. Table 2 shows that the main effect of time was significant, *F*(1, 60) = 55.163, *p* < 0.001, indicating that participants’ mean heart rate changed significantly from pre-test to post-test overall. The group-by-time interaction was also significant, *F*(2, 60) = 26.252, *p* < 0.001, partial *η^2^* = 0.467, indicating that the magnitude of pre-post change in mean heart rate differed significantly across the three groups. This result indicates that changes in heart rate differed across intervention conditions.

#### 3.2.3 Mean skin temperature

A linear mixed-effects model was established with mean skin temperature as the dependent variable. The results are shown in Table 2. Table 2 shows that the main effect of time was not significant, *F*(1, 60) = 1.476, *p* = 0.229. The group-by-time interaction was also not significant, *F*(2, 60) = 0.291, *p* = 0.748, partial *η^2^* = 0.010. These results indicate that the magnitude of change in mean skin temperature from pre-test to post-test did not differ significantly across the three groups. This result indicates that the skin temperature measure did not support significant physiological changes related to the intervention.

### 3.3 *Post hoc* comparisons of pre-post change scores between groups

#### 3.3.1 Academic anxiety score

Because the group-by-time interaction for academic anxiety scores was significant, *post hoc* comparisons were conducted to compare pre-post change scores across groups. The results are shown in Table 3. Table 3 shows that the decrease in academic anxiety was significantly greater in the LLM-VR-CBT group than in the Minimal-support control group, estimate = −3.55, 95% CI [−6.03, −1.07], *p* = 0.011. The decrease in academic anxiety was also significantly greater in the traditional CBT group than in the Minimal-support control group, estimate = −5.60, 95% CI [−8.08, −3.12], *p* < 0.001. The difference in pre-post change scores between the LLM-VR-CBT group and the traditional CBT group did not reach statistical significance, estimate = 2.05, 95% CI [−0.43, 4.53], *p* = 0.103.

**Table 3:** Post hoc comparison results for pre-post change scores between groups.

Outcome	Change-score comparison	Estimate	*SE*	*df*	*t*	95% CI	*p*
Academic anxiety	LLM-VR-CBT group–traditional CBT group	2.05	1.24	63.16	1.654	[−0.43, 4.53]	0.103
Academic anxiety	LLM-VR-CBT group–minimal-support control group	−3.55	1.24	63.16	−2.864	[−6.03, −1.07]	0.011
Academic anxiety	Traditional CBT group–minimal-support control group	−5.60	1.24	63.16	−4.518	[−8.08, −3.12]	<0.001
Mean heart rate	LLM-VR-CBT group–traditional CBT group	1.20	1.12	63.16	1.075	[−1.03, 3.43]	0.287
Mean heart rate	LLM-VR-CBT group–minimal-support control group	−6.15	1.12	63.16	−5.508	[−8.38, −3.92]	<0.001
Mean heart rate	Traditional CBT group–minimal-support control group	−7.35	1.12	63.16	−6.582	[−9.58, −5.12]	<0.001
Mean skin temperature	LLM-VR-CBT group–traditional CBT group	0.06	0.09	63.16	0.644	[−0.13, 0.25]	1.000
Mean skin temperature	LLM-VR-CBT group–minimal-support control group	0.06	0.09	63.16	0.644	[−0.13, 0.25]	1.000
Mean skin temperature	Traditional CBT group–minimal-support control group	0.00	0.09	63.16	0.000	[−0.19, 0.19]	1.000

Estimate represents the between-group difference in pre-post change calculated from estimated marginal means based on the LMM. Because this study used a balanced design, and because there were no missing data or additional covariates, the model-estimated within-group pre-post changes were numerically consistent with the post-pre changes calculated from the raw means in Table 1, with only possible differences due to rounding at the decimal level. Negative values indicate that the former group showed a greater decrease than the latter group. p values were adjusted using the Holm method for multiple comparisons.

#### 3.3.2 Mean heart rate

Because the group-by-time interaction for mean heart rate was significant, post hoc comparisons were conducted to compare pre-post change scores across groups. The results are shown in Table 3. Table 3 shows that the decrease in mean heart rate was significantly greater in the LLM-VR-CBT group than in the Minimal-support control group, estimate = −6.15, 95% CI [−8.38, −3.92], *p* < 0.001. The decrease in mean heart rate was also significantly greater in the traditional CBT group than in the Minimal-support control group, estimate = −7.35, 95% CI [−9.58, −5.12], *p* < 0.001. The difference in mean heart rate change between the LLM-VR-CBT group and the traditional CBT group did not reach statistical significance, estimate = 1.20, 95% CI [−1.03, 3.43], *p* = 0.287.

#### 3.3.3 Mean skin temperature

The group-by-time interaction for mean skin temperature was not significant, and further between-group comparisons of change scores also showed no significant differences. As shown in Table 3, the difference in skin temperature change between the LLM-VR-CBT group and the traditional CBT group was not significant, estimate = 0.06, 95% CI [−0.13, 0.25], *p* = 1.000. The difference between the LLM-VR-CBT group and the Minimal-support control group was not significant, estimate = 0.06, 95% CI [−0.13, 0.25], *p* = 1.000. The difference between the traditional CBT group and the Minimal-support control group was also not significant, estimate = 0.00, 95% CI [−0.19, 0.19], *p* = 1.000. This result further indicates that the skin temperature measure did not reflect significant intervention-related changes.

The intervention completion and available process records of the LLM-VR-CBT group are shown in Supplementary Table S2. This group had 80 planned LLM-VR-CBT sessions, and all 80 sessions were completed. It should be noted that safety-related events and system operation abnormalities were not systematically quantified as prespecified independent safety endpoints. Thus, this information is reported only as process records and as a study limitation.

### 3.4 Summary of results

In summary, the LMM results showed significant group-by-time interactions for academic anxiety and mean heart rate, indicating significant differences in the magnitude of pre-post change across intervention conditions. Further *post hoc* comparisons showed that the LLM-VR-CBT group and the traditional CBT group had significantly greater reductions in academic anxiety and mean heart rate than the minimal-support control group. This suggests that both active intervention conditions may have short-term positive effects on reducing academic anxiety and mean heart rate compared with the minimal-support control condition. The difference in change between the LLM-VR-CBT group and the traditional CBT group did not reach statistical significance. However, this result only indicates that no significant difference was observed in this study sample and cannot be interpreted as evidence that the two intervention conditions were equivalent. Mean skin temperature did not show a significant group-by-time interaction, indicating that the skin temperature indicator did not support a clear physiological regulation effect of the intervention. In addition, all planned sessions in the LLM-VR-CBT group were completed, and no missing primary outcome data resulted from incomplete sessions. However, safety-related events were not systematically collected as pre-specified independent endpoints, so these results cannot be used to infer the clinical safety or real-world feasibility of the system.

## 4 Discussion

This study preliminarily examined the short-term intervention effects of the LLM-VR-CBT system on academic anxiety among university students and compared it with traditional CBT and a minimal-support control condition. The results showed that the LLM-VR-CBT group and the traditional CBT group had significantly greater reductions in academic anxiety and mean heart rate than the minimal-support control group, suggesting that both active intervention conditions may have short-term positive effects. The difference in change between the LLM-VR-CBT group and the traditional CBT group did not reach statistical significance. However, because this study did not prespecify equivalence or noninferiority margins and did not conduct corresponding tests, this result only indicates that no significant difference was observed in this sample. It cannot be interpreted as evidence that the two intervention conditions had equivalent efficacy. Overall, this study provides preliminary evidence for the integrated LLM-VR-CBT intervention package as a digitally assisted intervention for academic anxiety among university students. It should be emphasized that the intervention evaluated in this trial was a multicomponent package combining an LLM-RAG-supported virtual agent, VR-based academic stress contexts, structured CBT modules, natural language interaction, and human supervision. Therefore, the observed effects should be interpreted as those of the integrated intervention package as a whole and cannot be attributed specifically to the LLM component.

From the perspective of existing research, the findings of this study are consistent with the general direction of digital CBT and technology-assisted psychological interventions. Previous studies suggest that internet-based CBT and digital CBT can provide some support for anxiety-related problems among university students and may help improve the accessibility of mental health services (11, 12, 40). VR exposure therapy and virtual agent interventions are also considered to enhance intervention experience through contextual simulation, interactive feedback, and embodied presentation (15, 20, 41). Compared with these studies, the main attempt of the present study was to integrate an LLM-RAG-supported virtual agent, VR-based academic stress contexts, and CBT intervention procedures into a structured system to support cognitive restructuring, virtual exposure, behavioral activation, and emotion regulation. The trial evaluated the combined effects of these components operating as an integrated system and did not use a component-controlled or dismantling design to isolate the independent contribution of each element. This system was not intended to replace traditional CBT. Instead, it explored how CBT techniques may be presented in a more contextualized, interactive, and individualized way in university academic stress settings through digital means.

The physiological indicator results further showed that both the LLM-VR-CBT group and the traditional CBT group had lower mean heart rate after the intervention, suggesting that active interventions may be related to short-term reductions in physiological arousal. However, skin temperature did not show a significant group-by-time interaction, and between-group comparisons of pre-post change did not reach statistical significance. Thus, the results of this study cannot be interpreted as a complete multimodal physiological recovery pattern. As a peripheral physiological indicator, skin temperature may be more easily affected by wearing position, ambient temperature, individual peripheral vascular responses, and short measurement duration. Future studies may combine indicators such as heart rate variability, electrodermal activity, and respiratory rate, and use longer measurement windows and stricter environmental control to further examine whether such interventions are associated with more stable changes in physiological arousal across multiple indicators.

From the perspective of system design, the LLM-VR-CBT system may support academic anxiety intervention through cognitive guidance, contextual exposure, and supportive interaction. The LLM-RAG-supported virtual agent can identify academic stress themes and negative automatic thoughts based on participants’ language input under predefined CBT procedures and knowledge-base constraints, and it can provide cognitive restructuring prompts and emotional support. VR scenarios provide relatively controlled simulated environments for stress contexts such as examinations, thesis writing, and classroom presentations. The virtual agent’s speech, facial expressions, and feedback behaviors may help enhance interaction continuity and task understanding. These design ideas are consistent with existing research directions on conversational agents, digital mental health interventions, and the potential of LLMs for personalized support (17, 18, 42, 43). However, it should be emphasized that this study did not directly measure process indicators such as the degree of cognitive restructuring, changes in avoidance behavior, self-efficacy, presence, satisfaction, engagement, module fidelity, or interaction quality. Therefore, the above mechanistic interpretations should be understood as theoretical assumptions based on system design and existing literature, rather than mediation mechanisms verified by this trial.

This study also has several limitations. At the study-design and reporting level, the sample came from a single university, the sample size was small, and the study examined only short-term changes after a 4-week intervention. The external validity and long-term stability of the findings therefore require further verification. The randomized trial was also not prospectively registered, which limits the transparency of the study protocol and the prespecification of outcomes and analyses. Future randomized trials should be registered in a public trial registry before participant enrolment. In addition, the minimal-support control group was not fully matched with the two active intervention groups in contact time, intervention expectations, level of research attention, or technological novelty. Therefore, between-group differences may have been partly influenced by nonspecific factors.

At the measurement and mechanism level, the Chinese AAS used as the primary outcome measure was translated and contextually adapted but was not independently psychometrically validated before the trial. Accordingly, the primary outcome findings should be interpreted cautiously, and future studies should formally validate the adapted version. Moreover, this study did not systematically measure process variables such as presence, usability, satisfaction, engagement, interaction quality, self-efficacy, or degree of cognitive restructuring. Thus, it could not examine specific mechanisms of action. Because the LLM-VR-CBT intervention was delivered as a multicomponent package, the study also could not isolate the independent contribution of the LLM, VR, CBT modules, embodied interaction, or human supervision.

At the system-evaluation and safety level, heart rate and skin temperature were used only as outcome indicators and safety observation references, and they did not constitute a real-time closed-loop regulation mechanism driven by physiological feedback. In addition, this study could report only intervention completion and partial process records. Adverse events, risk alerts, technical failures, human interventions, referrals, and protocol deviations were not systematically collected as prespecified independent safety endpoints. Therefore, the study cannot provide a sufficient judgment on the clinical safety or real-world feasibility of the system.

These limitations indicate several priorities for future research. Future studies should further examine the stability, safety, and application boundaries of the LLM-VR-CBT system using larger samples, multicenter designs, and long-term follow-up. Subsequent studies should prespecify a safety monitoring protocol and systematically record adverse events, risk alerts, technical failures, human interventions, referrals, and protocol deviations, in order to evaluate the safety and implementation boundaries of the LLM-VR-CBT system more rigorously. They should also include process indicators such as presence, usability, satisfaction, engagement, adherence, module fidelity, self-efficacy, and degree of cognitive restructuring. Combined with interaction logs and mediation analyses, these studies should examine whether system design features influence improvement in academic anxiety through user experience, engagement processes, or mastery of CBT techniques. After further safety evaluation, long-term follow-up, and real-world implementation studies are completed, this system should be further evaluated as a possible adjunctive support tool for early identification, psychoeducation, and low-intensity support for academic anxiety in university mental health services.

## 5 Conclusion

Within the theoretical framework of CBT, this study developed and preliminarily examined an LLM-VR-CBT system that integrates an LLM and VR to reduce academic anxiety among university students. The randomized controlled trial results showed that the LLM-VR-CBT group had significantly greater reductions in academic anxiety level and mean heart rate than the minimal-support control group. These findings suggest a possible short-term reduction in subjective academic anxiety accompanied by lower mean heart rate, but they do not establish a broad physiological regulation effect. At the same time, the difference in change between the LLM-VR-CBT group and the traditional CBT group did not reach statistical significance. However, because this study did not conduct formal equivalence or noninferiority testing, this result cannot be interpreted as evidence that the two intervention conditions had equivalent efficacy. Skin temperature did not show significant intervention-related changes, indicating that the physiological evidence was mainly concentrated on the heart rate indicator, and the related findings still need further verification.

Compared with existing studies, the main contribution of this study lies in integrating an LLM-supported virtual agent, RAG knowledge-base constraints, and VR-based immersive academic stress contexts into the CBT intervention procedure, and preliminarily evaluating its short-term intervention effects through a three-arm randomized controlled design. The system not only extends the application of virtual agents in academic anxiety intervention contexts, but also provides practical exploration for moving digital CBT interventions from fixed-script interaction toward more contextualized, personalized, and embodied forms of interaction. At the methodological level, this study combined subjective scale scores and physiological indicators to evaluate the effects of the digital intervention from multiple perspectives. At the design level, the system aimed to support a more contextualized, interactive, and flexible CBT-assisted intervention process through the virtual agent avatar, natural language interaction, cognitive restructuring guidance, and immersive exposure scenarios.

Overall, this study provides valuable preliminary evidence for the LLM-VR-CBT system as a digitally assisted intervention tool for academic anxiety among university students. It also provides new design ideas and a practical basis for introducing intelligent, contextualized, and interactive auxiliary intervention forms into university mental health services. Only after larger-sample studies, multicenter research, long-term follow-up, and predefined safety monitoring have been completed should such systems be further evaluated as adjunctive support tools for university mental health services.

## Data Availability

The original contributions presented in the study are included in the article/Supplementary material, further inquiries can be directed to the corresponding author.
