# Supplementary material for: Harnessing large language models in virtual CBT for university students’ academic anxiety: a preliminary randomized trial
Source: Front Public Health. 2026 Aug 27;14:1874822. doi: 10.3389/fpubh.2026.1874822 (PMC13558035; doi:10.3389/fpubh.2026.1874822)
Supplement: Supplementary file 1 [file Supplementary_file_1.pdf]

Appendix

Supplementary Table 1. Baseline characteristics of participants by group

Characteristic	LLM-VR-CBT group n = 20	Traditional CBT group n = 20	Minimal-support control group n = 20	Total N = 60
Age,*M (SD)*	20.85 (1.46)	20.90 (1.55)	20.86 (1.52)	20.87 (1.51)
Sex,*n* (%)				
Male	11(55.00)	10(50.00)	11(55.00)	32 (53.30)
Female	9(45.00)	10(50.00)	9(45.00)	28 (46.70)
Academic level, *n* (%)				
Undergraduate student	18(90.00)	18(90.00)	18(90.00)	54(90.00)
Graduate student	2(10.00)	2(10.00)	2(10.00)	6(10.00)
Major type, *n* (%)				
Science and engineering	7(35.00)	8(40.00)	7(35.00)	22(36.70)
Humanities and social sciences	7(35.00)	6(30.00)	7(35.00)	20(33.30)
Arts/design	6(30.00)	6(30.00)	6(30.00)	18(30.00)
AAS severity, *n* (%)				
Mild, 15–25 points	3 (15.00)	4 (20.00)	3 (15.00)	10 (16.70)
Moderate, 26–44 points	17 (85.00)	16 (80.00)	17 (85.00)	50 (83.30)
AAS total score, *M* (*SD*)	31.15 (5.64)	31.50 (5.82)	31.80 (4.89)	31.48 (5.38)
Mean heart rate, *M* (*SD*)	80.05 (4.82)	80.50 (4.49)	80.05 (5.11)	80.20 (4.74)
Mean skin temperature, *M* (*SD*)	30.18 (0.57)	30.11 (0.34)	30.04 (0.52)	30.11 (0.48)

Note: AAS = Academic Anxiety Scale. Continuous variables are presented as *M (SD)*, and categorical variables are presented as *n* (%). AAS severity was classified based on the baseline AAS total score, with 15 – 25 indicating mild anxiety and 26 – 44 indicating moderate anxiety.

Supplementary Table 2. Intervention completion and available process records in the LLM-VR-CBT group

Item	Recorded result
Planned LLM-VR-CBT sessions	80 sessions (20 participants × 4 sessions)
Completed LLM-VR-CBT sessions	80 sessions
Human supervision method	During LLM-VR-CBT sessions, researchers provided on-site or back-end supervision.
Expert review method	Two independent experts with CBT experience conducted quality reviews of randomly selected intervention audio recordings and dialogue logs. A fixed prespecified sampling proportion was not set as an independent quantitative indicator.

Note: This table summarizes only confirmable process records, including intervention completion, human supervision method, and expert review method. VR-related physical discomfort, physical or psychological adverse events, risk alerts for high-risk psychological content, the number of responses blocked or modified by safety rules, technical failures, human correction of agent responses, referrals to professional psychological support, and protocol deviations were not systematically quantified as prespecified independent safety endpoints. Therefore, this table is not used to estimate the incidence of adverse events and does not constitute sufficient validation of the clinical safety of the system.

Appendix 1. LLM-RAG Intervention System Technical Transparency Table

Note: This table is used to improve the descriptive technical transparency and process auditability of the LLM-RAG module in the LLM-VR-CBT system. It is not intended as a complete technical reproduction guide. This study recorded and controlled the system objective, CBT intervention modules, knowledge-base sources, prompt template constraints, safety rules, and human supervision procedures. Some lower-level retrieval and generation parameters used the default configuration of the AppBuilder platform. The retrieval number top-*k*, total number of knowledge-base documents, total number of chunks, and actual number of chunks generated for each document were not systematically recorded as experimental variables.

Item	Specific description
System objective	This system was used to assist academic anxiety intervention among university students. It provided support around academic stress identification,

	clarification of negative automatic thoughts, cognitive restructuring, emotion regulation, behavioral activation, and virtual exposure feedback. The system was positioned as an auxiliary digital intervention tool in addition to traditional psychological services, and was not used as an independent therapeutic agent.
Theoretical framework	The system was based on CBT as the theoretical framework. It focused on problems in academic anxiety, including catastrophic thinking, self-negation, expectations of failure, avoidance behaviors, procrastination behaviors, and emotional arousal.
Intervention modules	The system included four core modules: cognitive restructuring, virtual exposure, behavioral activation, and emotion regulation. The cognitive restructuring module was used to identify negative automatic thoughts and guide alternative cognitions. The virtual exposure module simulated academic stress contexts such as classroom presentations, examinations, and thesis defenses through VR. The behavioral activation module helped users break down learning tasks and develop plans. The emotion regulation module provided breathing training, mindfulness exercises, and stabilization strategies.
Base model and API version	The system used ERNIE-4.0-8K as the base LLM through the Baidu AI Cloud Qianfan AppBuilder platform. This model was mainly used for natural language understanding, dialogue generation, cognitive guidance, and emotional support. Model outputs were limited to academic anxiety support, CBT technique guidance, and general psychoeducation. The model did not directly generate psychiatric diagnoses, medication advice, or crisis intervention conclusions.
Public implementation reference	During system development, we referred to the public project “AI4CBT” on the ModelScope platform, which is related to CBT conversational agents. This project provides an example of fine-tuning a CBT dialogue model based on Qwen/Qwen2.5-7B-Instruct, including the LoRA fine-tuning method, training and validation dataset settings, system prompt, and model download method. It should be noted that this public project mainly served as a technical implementation reference for CBT conversational agents and was not equivalent to all deployment parameters of the final system in this study.
Reference fine-tuning configuration	The example configuration in the AI4CBT public project included the following: base model = Qwen/Qwen2.5-7B-Instruct; training method = LoRA; training data = train_.jsonl; validation data = valid_.jsonl; number of training epochs = 1; per-device train batch size = 1; per-device eval batch size = 1; learning rate = 1e-4; target modules = all-linear; gradient accumulation steps = 16; max_length = 4096; warmup_ratio = 0.05; dataloader_num_workers = 4; dataset_num_proc = 4. These parameters were used to describe the public fine-tuning implementation of a related CBT dialogue model and served as a reference for subsequent reproduction and extended development.

System prompt reference	The system prompt in the AI4CBT public project set the model as a “psychological counselor specializing in CBT.” It required the model to respond to users in a professional, patient, and compassionate way and to follow a CBT response structure, including validation and empathy, identification of key thoughts or beliefs, challenge or reflection, provision of strategies or insights, and encouragement and future orientation. This study referred to this CBT dialogue structure in the prompt template design, but further limited the system to academic anxiety support only. It did not provide diagnoses, medication advice, or crisis intervention conclusions.
Model calling method	The system called the LLM service through an API. After user input entered the back-end processing module through speech-to-text or text input, it went through problem identification, knowledge-base retrieval, and prompt template constraints. The LLM was then called to generate a response. After being checked by safety rules, model outputs were provided to users through text, speech, and virtual agent facial-expression feedback.
Platform and hosted resources	The agent and knowledge base were both built through the AppBuilder platform. The knowledge base used the default/shared hosted resources of the AppBuilder platform. The type of dedicated vector database instance was not separately recorded during the study.
Model calling parameters	The experimental system did not further tune generation parameters and mainly used the default parameters of the AppBuilder platform. The default chat parameters were temperature = 0.8, top_p = 0.8, and penalty_score = 1.0. The default thinking parameters were temperature = 0.01, top_p = 0, and penalty_score = 1.0. These parameters were not manipulated as experimental variables.
Virtual agent presentation	The virtual human avatar was generated using MetaHuman Creator and imported into the Unreal Engine 5 scene through Quixel Bridge. The character was designed as a gentle and professional psychological instructor. The system enhanced the sense of embodied interaction through facial expressions, head orientation, nodding feedback, moderate gestures, and posture changes. Speech interaction followed the workflow of “user speech input—speech-to-text—LLM text generation—text-to-speech output,” allowing participants to interact with the virtual agent in a relatively natural conversational manner.
RAG architecture	The system adopted a RAG architecture. User input was first identified as a specific problem type. The system then retrieved relevant content from the knowledge base, used the retrieved content as contextual input for the prompt template, and generated a response through the LLM. This process was used to constrain the model’s response scope and reduce the risk of hallucination and deviation from CBT principles.
Embedding model and vectorization	The knowledge base used the AppBuilder platform’s default vector model, multilingual embedding AppBuilder. Materials uploaded to the knowledge base were parsed, chunked, and vectorized through the AppBuilder

	knowledge-base workflow. This study did not compare the embedding model as an experimental variable.
RAG retrieval settings	The system used the AppBuilder knowledge-base retrieval workflow. Under the customized chunking setting, the current default maximum chunk length was 1200 Chinese characters. During the study, the total number of knowledge-base chunks, the actual number of chunks for each document, the top-*k* retrieval number, and the matching threshold were not exported or systematically recorded. Therefore, these parameters are reported as platform default or unrecorded parameters, rather than controlled reproducible experimental parameters.
Knowledge-base sources	The knowledge base mainly included three types of materials. First, it included theories, scales, and intervention studies related to academic anxiety. Second, it included CBT counseling technique materials, including cognitive restructuring, behavioral activation, exposure training, emotion regulation, time management, and problem-solving training. Third, it included dialogue materials developed based on expert interviews and common academic stress scenarios among university students.
Knowledge-base size	The knowledge base mainly consisted of theories, scales, and intervention studies related to academic anxiety, CBT counseling technique materials, and dialogue materials developed based on expert interviews and common academic stress contexts. During the study, the total number of source documents in the knowledge base, total number of chunks, and actual number of chunks generated from each document were not systematically exported.
Dialogue corpus sources	The dialogue corpus mainly came from three sources. The first source was common expressions of academic anxiety identified from previous user interviews, such as concerns about exam failure, thesis writing pressure, classroom presentation anxiety, procrastination, and self-negation. The second source was common CBT counseling guidance language extracted from expert interviews. The third source was simulated dialogue samples written by the research team based on typical academic stress contexts and reviewed by experts. All personal identity information was removed before the corpus entered the knowledge base.
Dialogue corpus annotation scheme	The dialogue corpus was structurally annotated according to "academic stress context—main negative automatic thought—corresponding CBT technique—recommended response strategy—risk level." Typical contexts included thesis writing pressure, exam anxiety, classroom presentation anxiety, procrastination, and self-negation. Risk level was used to distinguish general academic anxiety support content from content requiring professional referral or crisis escalation.
Knowledge-base inclusion criteria	Materials included in the knowledge base had to meet the following criteria: clear source; direct relevance to academic anxiety or CBT intervention; language suitable for university students; ability to be transformed into structured intervention language; and no unverified high-risk clinical advice,

	medication advice, or diagnostic conclusions.
Knowledge-base exclusion criteria	Materials were excluded if they contained psychological advice without source support, unverified treatment methods, content that might induce self-diagnosis or medication use, expressions that might increase anxiety or self-blame, or automated advice related to self-harm, suicide, or severe psychiatric symptoms without professional human intervention.
Knowledge-base processing workflow	Knowledge-base materials entered the system after collection, deduplication, anonymization, thematic classification, expert review, and structured organization. Each knowledge fragment needed to be annotated with its corresponding module, such as cognitive restructuring, virtual exposure, behavioral activation, emotion regulation, time management, or risk referral.
Prompt template design	The prompt template included five types of constraints: role setting, task objective, CBT principles, response boundaries, and safety rules. The model was set as an "academic anxiety support virtual agent." Its responses were required to follow the principles of supportiveness, nonjudgment, evidence-based guidance, conciseness and clarity, and nonreplacement of professional counselors.
Representative system prompt content	The prompt template defined the virtual agent as an "academic anxiety support virtual agent." It required the agent to respond to users in a gentle, professional, supportive, and nonjudgmental manner. It prioritized CBT techniques to help users identify negative automatic thoughts, examine evidence, form alternative thoughts, and develop executable action plans. It was not allowed to provide psychiatric diagnoses, medication advice, or crisis intervention conclusions. When self-harm, suicidal ideation, severe depression, or other high-risk expressions were identified, the system was required to stop automated intervention and suggest that the user contact the campus mental health center or professional staff.
Hallucination control strategies	The system reduced hallucination risk through RAG retrieval, prompt template constraints, response scope limitation, risk-word checking, and human spot checks. For questions beyond the scope of the knowledge base, the system was required to avoid fabricating professional conclusions and to suggest that users consult the campus mental health center or professional staff.
Interaction workflow	The basic workflow was as follows: user input identification—knowledge-base retrieval—prompt template constraint—LLM generation—safety rule review—virtual agent output. Output formats included speech, text, facial expressions, and simple nonverbal feedback.
API calling workflow	The system calling workflow can be summarized as follows: ① receive user speech or text input; ② transcribe speech input into text; ③ identify the user's problem type and risk level; ④ retrieve the CBT knowledge base according to the problem type; ⑤ combine the retrieval results, user input, and prompt template into model input; ⑥ call the LLM API to generate a response; ⑦ check the response using safety rules; ⑧ output the response

	through text, speech, and virtual agent movements; and ⑨ record module calls, usage duration, and key dialogue content.
Boundary of physiological indicator use	The system collected heart rate and skin temperature as physiological outcome indicators and auxiliary assessment indicators to evaluate changes in physiological arousal before and after the intervention. Heart rate and skin temperature were not input into the LLM-RAG module, did not participate in real-time module selection, were not used to automatically trigger the emotion regulation module, and were not used to adjust exposure task difficulty. System module calls were mainly based on user speech or text input, subjective anxiety feedback, the current intervention module, risk-word identification results, and human supervision procedures.
Human supervision mechanism	The system backend recorded the completion status, usage duration, module call sequence, and key dialogue content for each intervention. Two independent experts with CBT experience conducted quality reviews of randomly selected audio recordings and dialogue logs to assess whether they followed CBT principles, deviated from intervention goals, or contained potentially inappropriate responses.
Intervention fidelity control	The LLM-VR-CBT group completed the intervention according to the predefined module and time structure. Each intervention session included a 5-min emotional warm-up, a 40-min core intervention, and a 5-min summary and feedback phase. Expert spot checks were used to confirm whether system outputs covered the target modules and followed the basic procedures of cognitive restructuring, exposure training, behavioral activation, and emotion regulation.
Psychological risk identification	The system identified self-harm, suicidal ideation, severe depression, psychotic symptoms, expressions of extreme hopelessness, and other high-risk content. Once high-risk content appeared, the system no longer provided automated CBT intervention.
Risk escalation procedure	When users expressed high-risk psychological content, the system generated a crisis prompt, suggested that users immediately contact the campus mental health center, a professional psychological counselor, or emergency help resources, and marked the record as requiring human review. The research team conducted human confirmation and necessary referral according to the risk level.
Privacy and data protection	All participants signed informed consent before the experiment. System records were used only for research analysis, intervention quality review, and safety monitoring. Data were anonymized before analysis, and research team members accessed relevant data only within the authorized scope.
System boundaries	This system served only as a digitally assisted intervention tool for academic anxiety. It did not provide psychiatric diagnosis, medication treatment advice, crisis intervention conclusions, or substitute psychotherapy. Severe psychological risks or complex clinical problems should be referred to professionals.

Training and deployment boundaries	The training parameters in the AI4CBT public project can be used to describe the fine-tuning method of a related CBT dialogue model. However, this study did not treat the full training configuration of that public project as the direct source of the system's intervention effects. This study mainly evaluated the overall effects of the LLM-VR-CBT intervention system embedded in VR scenarios, constrained by the RAG knowledge base, and supported by human supervision procedures.

Algorithm S1. LLM-RAG Intervention Response Generation Workflow

Input: user speech or text input, current intervention module, user subjective status records, and risk identification rules
Output: virtual agent response, module invocation records, and safety review results
Note: Heart rate and skin temperature data were not used as input variables for this algorithm. They did not participate in virtual agent response generation, module invocation, or exposure task difficulty adjustment. They were used only for pre-post physiological outcome assessment and research recording.

1. Receive user input and transcribe voice input into text.
2. Identify the problem type in the user text and determine whether it belongs to test anxiety, thesis pressure, class presentation anxiety, procrastination, self-negation, or other types of academic stress.
3. Conduct psychological risk screening on the user text and determine whether it contains self-harm, suicidal ideation, severe depression, psychotic symptoms, or other high-risk content.
4. If high-risk content is identified, stop automated intervention, generate a crisis alert, and mark the record for human review.
5. If no high-risk content is identified, generate a retrieval query according to the problem type.
6. Retrieve relevant knowledge snippets from the CBT knowledge base and the academic anxiety corpus.
7. Combine the user input, retrieval results, current module objective, and prompt template into the model input.
8. Invoke the LLM API to generate a candidate response.
9. Conduct a safety rule check on the candidate response, and delete or modify diagnostic, medication-related, absolutist, or high-risk suggestions.
10. Output the final response through text, speech, and virtual agent facial expressions.
11. Record the module type, duration of use, key dialogue content, and risk flag for the current interaction for later expert spot checks.

Example Interaction Workflow

Stage	Example content
User input	"I feel very anxious whenever I think about my thesis recently. I always feel that I definitely cannot write it well, and that my advisor will also think I am not capable."
Problem identification	The system identifies the input as thesis-writing-related academic anxiety and detects negative automatic thoughts, including catastrophic prediction and self-negation.
Knowledge base retrieval	The system retrieves CBT content related to cognitive restructuring, thesis task breakdown, academic self-efficacy, and emotional stabilization strategies.
System response	"It sounds like you are directly linking the outcome of your thesis with your personal ability, which can make the anxiety feel stronger. We can first break this thought down: Does 'I cannot write it well' necessarily mean 'I am not capable'? Are there other possibilities, such as the task being too large, the time plan being unclear, or you have not yet broken the thesis into smaller steps? Next, we can first list one small task that can be completed today, such as organizing one reference or writing an outline for one paragraph."
Intervention module	Cognitive restructuring + behavioral activation
Safety boundaries	The response does not provide diagnosis or medication advice. It only provides academic stress support, cognitive clarification, and task breakdown.

Mental Health Risk Escalation Workflow

Risk level	User expression type	System processing method
Low risk	General academic anxiety, procrastination, exam worries, and thesis pressure	Continue to provide CBT technique guidance, such as cognitive restructuring, task breakdown, emotion regulation, and exposure practice.
Medium risk	Obvious persistent low mood, strong helplessness, severe sleep problems, or functional impairment	Advise the user to contact the campus mental health center or a professional counselor, and prompt the research team to conduct human review.
High risk	Self-harm, suicidal ideation, extreme despair, psychotic symptoms, or other crisis content	Immediately stop automated intervention, generate a crisis alert, advise the user to contact the campus mental health center, professionals, or emergency support resources, and mark the record as requiring human review and referral.

Appendix 2. Academic Anxiety Scale (AAS)

Name:

Sex:

Grade:

Dear student,

Please answer the following questions using the four-point scale below according to your academic anxiety. There are no right or wrong answers. Please answer honestly. Thank you for completing this questionnaire.

1 = Completely inconsistent; 2 = Slightly consistent; 3 = Relatively consistent; 4 = Completely consistent

Scoring criterion: In this study, a total score ⩾15 was used as the screening criterion for evident academic anxiety. This criterion was used for study inclusion and severity stratification, and was not used as a clinical diagnostic basis.

Scale source: https://sites.bsu.edu/aarc/research/academic-anxiety-scale/

**No.**	**Item**	**1**	**2**	**3**	**4**
1	I often worry that my school performance will not be as good as I expect.	1	2	3	4
2	Because schoolwork gives me stress, I tend to put off doing schoolwork.	1	2	3	4
3	I often worry that I have not done my assignments well.	1	2	3	4
4	I am less confident at school than my classmates.	1	2	3	4
5	When I am in the classroom, I have a sense of fear.	1	2	3	4
6	I think my teachers are frightening.	1	2	3	4
7	I spend most of my time at school worrying about what to do next.	1	2	3	4
8	Some things at school make me feel afraid.	1	2	3	4
9	I am very worried about what my classmates think of my abilities.	1	2	3	4
10	When I need to do important classroom assignments, I often feel uncomfortable.	1	2	3	4
11	I find it difficult to take on responsibilities at school.	1	2	3	4

Methodological note: This appendix presents the complete AAS version actually administered in this study. Formal screening, anxiety severity stratification, stratified randomization, and primary

outcome statistical analysis were all based on the 11 items listed in this appendix. Each item was scored from 1 to 4. The scores of the 11 items were directly summed to produce the total score, with a total score range of 11–44. A total score ≥15 was used as the screening criterion for evident academic anxiety. This criterion was used only for study inclusion and severity stratification, and was not used as a clinical diagnostic basis. This study did not delete, merge, or replace any item. The AAS total score used in the statistical analysis corresponded exactly to the 11-item version listed in this appendix.

Appendix 3. Interview Guide Content

User Interview Guide

**Interview dimension**	**User interview questions**
Basic user information	Year/grade, major, and sex.
Basic user information	Have you experienced obvious academic anxiety in the past year?
Current status of academic anxiety assessment	Please describe the type of academic anxiety that you have experienced most recently or most frequently, such as test anxiety, task procrastination anxiety, or grade comparison anxiety. Please also give examples of specific trigger scenarios.
Current status of academic anxiety assessment	What specific effects has academic anxiety had on your study and daily life?
Current status of academic anxiety assessment	When you feel academic anxiety, how do you usually relieve it? What do you think are the limitations of your current coping methods? Have you used VR or similar technologies to support learning or relaxation?
Preferences for immersive sensory experience elements	In what type of virtual environment would you prefer to receive anxiety intervention, such as a counseling room, study, or natural environment?
Preferences for immersive sensory experience elements	What level of anthropomorphism would you prefer for the agent? What expectations do you have for the appearance of a virtual counselor?
Preferences for immersive sensory experience elements	If the agent responds to your emotions through facial microexpressions or body movements, do you think this would enhance the realism of the dialogue?
Preferences	How do you view the virtual agent's use of body language, facial expressions, and

for immersive sensory experience elements	posture during communication? Which aspects are especially important to you? For tone of voice and speech style, what style would you prefer?
Preferences for interaction experience elements	Through which mode would you prefer to interact with the system?
Preferences for interaction experience elements	What aspects of data privacy concern you most?
Preferences for interaction experience elements	If the system needs to record your academic behavior patterns to provide personalized recommendations, what range of data collection would you find acceptable?
Preferences for interaction experience elements	Would you be willing to complete questionnaires or data collection at the initial stage to help the system better assess your situation?
Preferences for interaction experience elements	When the system helps you set personalized intervention goals, what kind of goal-setting process would you prefer, such as joint discussion or a combination of system recommendations and user self-selection?
Preferences for interaction experience elements	For a task-oriented interaction mode, what forms of tasks would you prefer, such as scenario simulation or emotion expression exercises?
Preferences for interaction experience elements	When you encounter academic problems, what kind of problem analysis and problem-solving approach are you more accustomed to, such as independent thinking, discussion with others, or reliance on professional advice?
Preferences for interaction experience	How do you view periodic assessment feedback and immediate feedback after task completion? Which form of feedback would better help you see your progress, such as progress visualization, achievement badges, or anxiety reports?

elements	
Preferences for affective experience elements	How should the system express care and understanding through language style and communication style so that you feel supported?
Preferences for affective experience elements	When negative thoughts or emotional fluctuations occur, how would you like the system to help you identify and regulate your emotions?

Expert Interview Guide

Interview dimension	Expert interview questions
Basic expert information	Professional background and work experience.
Basic expert information	In which areas is your use of CBT methods and experience in academic anxiety intervention mainly concentrated? Have you had specific practical cases involving university students?
Current status of academic anxiety assessment	What do you think are the main manifestations of academic anxiety? Which types of academic anxiety have the most significant effects on university students?
Current status of academic anxiety assessment	In your view, what roles can a virtual agent system play in intervening in academic anxiety? Is it suitable for handling different dimensions of academic anxiety? What factors need to be considered in the design of the interaction mechanism?
Privacy protection mechanism	For privacy protection, what data collection and privacy protection mechanisms do you think experts are most concerned about in CBT intervention?
Anxiety assessment	In CBT intervention, how can effective assessment tools, such as questionnaires and interviews, help students identify the core problems of academic anxiety? Which factors do you think are especially important in the assessment stage?
Intervention goal setting	During the goal-setting process, how can intervention goals be made personalized and achievable? What do you think about the effects of dynamically adjusting goals for students?
Cognitive restructuring	What role does cognitive restructuring play in academic anxiety intervention? How do you think students can be helped to identify and challenge academic-related negative automatic thoughts?
Behavioral activation and exposure	In a virtual system, how should task breakdown and exposure scenarios be designed to enhance engagement among students with different types of academic anxiety?
Problem-solving skills training	In CBT intervention, what is the focus of problem-solving skills training? Which strategies do you think can best help students find feasible solutions when facing academic challenges?
Maintenance and consolidation	During academic anxiety intervention, what kind of feedback mechanism do you think can help students identify their progress and motivate them to continue participating in the intervention? How should the system provide follow-up support after the intervention ends?
Summary	What are your views on the development prospects of virtual agent systems in academic anxiety intervention?

Appendix 4. Dataset Content Examples

Cognitive Behavioral Therapy Dataset

Source： https://www.modelscope.cn/models/daydreamertc/AI4CBT/summary

Academic Anxiety Domain Knowledge Dataset Example

1. Manifestations of Academic Anxiety

Emotional manifestations:

Anxiety, tension, irritability, and fear. Students may feel panic about upcoming examinations or academic tasks. They may have excessive worries about failure or low grades and often believe that they are unable to cope with academic pressure. Their emotions may be unstable. They may become angry easily, lose control, or fall into depressive moods.

Physiological manifestations:

Physiological symptoms such as insomnia, headache, stomachache, and loss of appetite may occur. Frequent anxiety responses may also appear, such as sweating, accelerated heartbeat, and shortness of breath. Students may experience a serious lack of energy and feel tired or weak.

Cognitive manifestations:

Students may have difficulty concentrating, confused thinking, and a tendency to experience a “blank mind.” They may also have excessive worries about examinations and learning tasks and may worry that they cannot complete tasks or may fail.

Behavioral manifestations:

Students may strongly avoid academic-related activities and may procrastinate or avoid learning tasks. They may also overprepare by spending a large amount of time reviewing and preparing, without showing clear improvement. They may be highly sensitive to grades, frequently check grades, or show a strong tendency toward perfectionism.

2. Specific Contexts of Academic Anxiety

The triggering contexts of academic anxiety are not caused by a single factor, but are the result of multiple interacting factors. The following are different types.

1. Personal growth type

Self-identity confusion:

University students are in a critical stage of personal growth. Especially when academic pressure is high, they may easily experience self-doubt and anxiety, question their own ability, and feel uncertain about whether their goals are clear.

Perfectionistic tendency:

Some students pursue perfection and believe that they must perform best in all academic tasks. This perfectionistic mindset can easily lead to anxiety.

Competitive pressure:

In a competitive academic environment, some students feel expectations from themselves or from others. They may experience anxiety especially when facing high-performing peers.

2. Academic stress type

Examination or grade pressure:

When facing upcoming final examinations, semester grade evaluations, or academic reviews, students often experience intense emotional pressure and worry that they cannot meet expectations.

Accumulation of long-term pressure:

Long-term accumulation of academic tasks, assignments, projects, and examination pressure may lead to excessive emotional tension or breakdown, further intensifying anxiety.

Emotional instability:

Poor emotion management may make students easily fall into a cycle of anxiety. For example, when facing difficulties or challenges, they may experience excessive anxiety and worry, or even feel unable to control their emotions.

3. Academic education type

Heavy and urgent academic tasks:

A large number of course assignments, projects, papers, and upcoming examinations can make students feel unable to cope. Anxiety may intensify especially when tasks accumulate.

Inappropriate learning methods or self-doubt:

If students fail to find suitable learning methods, or if they doubt their own learning ability, they may often feel self-blame or anxiety.

Maladjustment to the academic environment:

For example, in cross-disciplinary learning, when course content is too difficult or course requirements are too demanding, students may feel anxious because they cannot adapt.

Academic failure:

When students fail to complete an academic task as expected or fail to achieve an ideal grade, they may easily fall into self-negation, anxiety, or even depression.

3. Coping Strategies for Academic Anxiety

1. Cognitive adjustment strategies

Restructuring negative thinking:

Academic anxiety often stems from excessive worries about future outcomes and denial of one’s own ability. Through cognitive restructuring, students can be helped to reassess and adjust these negative beliefs.

Setting realistic goals:

Students should set specific and achievable academic goals according to their abilities and avoid setting overly high expectations. Breaking large tasks into smaller and manageable parts helps reduce anxiety.

Changing views of failure:

Academic failure can be viewed as part of learning and growth, rather than as a denial of self-worth. Students can learn to draw lessons from failure, rather than allowing failure to bring persistent anxiety.

2. Emotion management strategies

Emotional expression and release:

Students are encouraged to express and release their emotions. They can reduce anxiety and emotional suppression through journaling, talking with friends, or artistic creation.

Self-compassion:

Students can learn self-compassion and accept their vulnerability and imperfection when facing pressure. They can give themselves space to feel and accept their anxiety, rather than suppressing it.

Emotion regulation skills:

Students can regulate emotions through meditation, deep breathing, or relaxation training. For example, deep breathing exercises can help reduce physical tension and improve anxious mood.

3. Time management strategies

Developing a reasonable timetable:

To cope with academic pressure, students need to arrange their time reasonably and develop feasible study and rest plans. They should ensure that they have enough time each day to complete academic tasks, while also arranging rest and leisure time to avoid overwork.

Prioritizing tasks:

Tasks can be ranked according to urgency and importance, with high-priority tasks handled first. Time management tools, such as the Pomodoro Technique, schedules, or to-do lists, can be used to help allocate time.

Avoiding procrastination:

Procrastination is an important source of academic anxiety. Students can gradually complete tasks by setting small, short-term goals and avoid being overwhelmed by the pressure of large tasks.

4. Interpersonal support strategies

Seeking social support:

Students can talk about their anxiety with family members, friends, or classmates, share pressure,

and obtain support. Social support can relieve stress and increase psychological resilience.

Building healthy social relationships:

Participating in social activities and group study can not only help improve academic performance, but also reduce loneliness and anxiety.

Seeking professional help:

If anxiety is difficult to regulate independently, students can seek help from psychological counselors or academic advisors to obtain professional emotion management and academic guidance.

5. Relaxation and recovery strategies

Exercise and physical activity:

Physical activities such as walking, swimming, and yoga not only improve physical health, but may also significantly reduce anxiety levels. Regular exercise helps release endorphins, improve mood, and reduce academic pressure.

Adequate sleep:

Insufficient sleep can lead to poor concentration and aggravated anxiety. Therefore, ensuring 7-9 hours of high-quality sleep each day is very important. Good daily routines help restore energy and maintain psychological health.

Relaxation techniques:

Students can practice techniques such as progressive muscle relaxation, meditation, or visualization-based relaxation to help relax the body and reduce the negative effects of academic pressure.

6. Self-motivation strategies

Reward system:

Students can set small goals and give themselves appropriate rewards after achieving them, such as watching a movie, eating a favorite food, or taking a rest. Positive reinforcement can help maintain learning motivation.

Focusing on the process rather than the outcome:

Students can shift their attention from the final result to the learning process and enjoy learning itself, rather than being overly concerned with achievement. This can help reduce anxiety and increase intrinsic motivation.

Cognitive Behavioral Therapy Dataset Dialogue Examples

Dialogue Example 1

Client: Recently, I have felt very anxious, but I do not know how to relieve it.

Counselor: I understand how you feel. This feeling of anxiety must make you feel uneasy, right? Anxiety is actually a normal emotion. It reminds us to pay attention to important things. However, when it becomes too intense and affects our daily life, we need to find more effective ways to cope with it.

Client: In my studies, I always feel that I am not doing well enough. No matter what grades I get, I cannot truly feel satisfied. Whenever I face a task, I feel intense anxiety. I worry that my efforts are not perfect enough, and I am even afraid of failure. This pursuit of perfection makes me feel burdened, as if only doing the best would mean that I have not failed.

Counselor: It sounds like you have very high standards for your performance, and when you evaluate your own value, it may be closely tied to these standards. I would like to ask you: if one day you do not meet these perfect standards, how would you view yourself?

Client: I would feel that I am not good enough. I might even doubt whether I am not working hard enough or whether I am simply not suited to this field.

Counselor: I understand. This kind of thought may make you more self-critical. Have you noticed that this thinking pattern may involve "black-and-white thinking" or "catastrophic thinking"? In other words, you may be viewing success and failure as two extremes, without considering the value of effort and progress themselves. Could we try to look at "success" from another perspective? For example, could success also include growth and learning, rather than only a perfect outcome?

Client: But I always find it difficult to concentrate, and I also procrastinate on tasks, which makes the pressure greater and greater.

Counselor: Procrastination is indeed a way many people respond to pressure. To some extent, it may be a strategy to avoid failure. However, in the long term, this strategy may make anxiety more serious. Would you be willing to try a small adjustment, such as breaking tasks into smaller parts and setting a feasible timetable? In this way, each time you complete a small task, you may gain a sense of control, instead of facing a large task that feels difficult to begin.

Client: But I feel very tired every day, and it is difficult to find the motivation to make these changes.

Counselor: I hear that you are being honest about your feelings. Fatigue and lack of motivation do influence each other. Your brain and body may both be sending you signals, reminding you that you need rest and balance. Do you have a regular schedule and rest time? Sometimes, appropriately adjusting sleep, diet, and exercise can help us improve energy and gradually regain a sense of control over tasks. Would you be willing to try adjusting your daily rhythm?

Client: Actually, I am also afraid that I will never be able to get rid of anxiety.

Counselor: I can understand your concern. When facing anxiety, we often feel that it is uncontrollable. But please remember that anxiety will not last forever, and we can regulate it through strategies. You can try some methods, such as mindfulness practice, deep breathing techniques, or writing down your anxious feelings, to help you observe your thinking patterns more clearly. At the same time, communicating with trusted people and seeking support are also very important. Would you be willing to try some of these methods?

Client: I am willing to try. Thank you for your support and understanding.

Counselor: You have already taken an important step, and that itself deserves recognition. Please remember that anxiety is not the endpoint. I believe that you have the ability to face it, and you are not alone. If you need support, I will be here for you.

Dialogue Example 2

Client: I have been feeling very down recently.

Counselor: I hear how you feel. This must be difficult for you, right? Feeling down is a common emotion. It may come from unmet expectations, low self-evaluation, or uncertainty about the future. You are not alone. Many people go through moments like this. What matters is that we can explore some methods together to help you gradually ease this emotion.

Client: Okay. What methods would you recommend?

Counselor: Several methods may help us gradually adjust our mindset. One is “behavioral activation,” which means taking action first rather than waiting for emotions to improve. For example, completing one small thing, such as tidying your room, taking a walk, or doing an activity you enjoy, may bring a sense of achievement and positive feedback. Another method is “cognitive restructuring.” This involves adjusting our evaluations of ourselves and reducing excessive self-criticism, such as actively identifying areas where we have done well and giving ourselves positive self-affirmation. Would you be willing to try one of these methods?

Client: These methods sound good, but I do not know how to do them in practice.

Counselor: That is very normal. We can take it step by step. You can first choose one small and specific thing, such as completing one small task each day, tidying your desk, or writing down one thing you completed today and giving yourself affirmation. You can observe whether you feel slightly better after completing these tasks. At the same time, you can try sharing your efforts and progress with friends or family members around you, so that their positive feedback becomes part of your motivation.

Client: Thank you for the advice, but I am still a little afraid of failure.

Counselor: I understand your concern. The fear of failure feels very real, especially when we

equate failure with a lack of personal ability. However, failure is essentially part of learning and growth. If we can view failure as an opportunity for adjustment and improvement, rather than as a denial of personal value, it may become less frightening. Would you be willing to recall a past failure you experienced and consider whether it eventually brought you some kind of learning or growth?

Client: Yes, some failures did teach me things, but they were still difficult to accept at the time.

Counselor: That is very natural. Failure can indeed bring discomfort in the moment, but we can learn to view it from a longer-term perspective. You can try asking yourself, “If this experience happened to my friend, how would I encourage them?” This method can help you view yourself from a gentler and more objective perspective.

Client: I understand. Thank you for your support.

Counselor: You are welcome. I would also like to share another interesting method with you: the “helping others” method. When we encourage others and acknowledge their efforts, we can also gain satisfaction and confidence from the process. For example, you can try encouraging your friends, sincerely acknowledging their efforts, and giving them positive feedback. This can not only help them build confidence, but also allow you to experience positive emotional feedback in the process. Would you be willing to try it?

Client: This method sounds like it could bring a sense of achievement. I will try it.

Counselor: That is great. Please remember that every attempt and every effort is a step in a positive direction. If you encounter difficulties in this process, we can adjust the method at any time. You are already on the path of making changes, and I am proud of you.

Appendix 5. Model Performance Evaluation Questionnaire

Dear expert, thank you very much for taking the time to participate in this model evaluation. In this evaluation, we will show you responses generated by each model in multiple academic anxiety contexts. Please evaluate the model-generated responses according to the criteria below in terms of comprehensiveness, professionalism, realism, and privacy protection. Higher scores indicate better performance.

**Dimension**	**Description**	**Criterion**	**Criterion score**	**Dimension score**
Comprehensiveness	Evaluate whether the dialogue comprehensively reflects the client's situation and mental health problems.	1.1 Does the dialogue reflect the client's basic information?	1	2
Comprehensiveness	Evaluate whether the dialogue comprehensively reflects the client's situation and mental health problems.	1.2 Does the dialogue reflect the client's mental health problems?	1	
Professionalism	Evaluate the professional competence demonstrated by the counselor in the dialogue.	2.1 Does the counselor demonstrate professional competence in diagnosing mental health problems?	0.5	4
Professionalism	Evaluate the professional competence demonstrated by the counselor in the dialogue.	2.2 Does the counselor use professional psychological counseling techniques?	0.5	
Professionalism	Evaluate the professional competence demonstrated by the counselor in	2.3 Is the counselor's language professional, and does the dialogue	0.5	

	the dialogue.	include guided questioning?		
Professionalism	Evaluate the professional competence demonstrated by the counselor in the dialogue.	2.4 Does the dialogue reflect the client's counseling purpose?	0.5	
Professionalism	Evaluate the professional competence demonstrated by the counselor in the dialogue.	2.5 Does the dialogue follow the sequence of a professional counseling framework, including the reception and inquiry stage, diagnostic stage, counseling stage, and consolidation and closing stage?	1	
Professionalism	Evaluate the professional competence demonstrated by the counselor in the dialogue.	2.6 Do the psychological counseling techniques have specific implementation procedures that are as detailed and clear as possible?	1	
Realism	Evaluate whether the dialogue is consistent with a real psychological counseling context.	3.1 Does the client express emotions and emotional changes that are appropriate to the scenario?	1	3
Realism	Evaluate whether the dialogue is consistent with a real psychological	3.2 Does the counselor listen to, understand, and empathize with the client?	0.5	

	counseling context.			
Realism	Evaluate whether the dialogue is consistent with a real psychological counseling context.	3.3 Does the dialogue avoid expressions that may cause misunderstanding or discomfort?	0.5	
Realism	Evaluate whether the dialogue is consistent with a real psychological counseling context.	3.4 Does the dialogue avoid lengthy statements, and is it consistent with a real psychological counseling context?	1	
Privacy protection	Ensure that the dialogue protects the client's privacy.	4.1 Does the dialogue comply with psychological counseling privacy guidelines and avoid revealing sensitive information, such as personal name, workplace, contact information, and home address?	0.5	1
Privacy protection	Ensure that the dialogue protects the client's privacy.	4.2 Does the dialogue respect the client's thoughts and emotions?	0.5	

Note: The questionnaires listed in this appendix were used during the system development and pre-evaluation stage to help optimize the virtual agent's response quality, usability, and immersive experience design. These questionnaires were not prespecified outcome measures of this randomized trial, nor were they statistically analyzed as formal outcome variables among the 60 randomized participants. Therefore, these materials are provided only as supplementary information for system development transparency and are not used as empirical evidence for the intervention effects or mechanisms of action in this study.

Appendix 6. Usability and Presence Test Evaluation Questionnaire

Dear student, welcome to this usability and presence test. Please complete the scales according to how you feel at this moment. Your responses will provide reference for subsequent design. All your information will be kept strictly confidential. Thank you for your participation.

System Usability Scale(SUS)

No.	Item	Strongly disagree	Disagree	Neutral	Agree	Strongly agree
1	I think I would like to use this system frequently.	1	2	3	4	5
2	I found the system unnecessarily complex.	1	2	3	4	5
3	I thought the system was easy to use.	1	2	3	4	5
4	I think that I would need the support of a technical person to be able to use this system.	1	2	3	4	5
5	I found the various functions in this system were well integrated.	1	2	3	4	5
6	I thought there was too much inconsistency in this system.	1	2	3	4	5
7	I imagine that most people would learn to use this system very quickly.	1	2	3	4	5
8	I found the system very cumbersome to use.	1	2	3	4	5
9	I felt very confident using the system.	1	2	3	4	5
10	I needed to learn a lot of things before I could get going with this system.	1	2	3	4	5

Presence Questionnaire(Scoring range: -3, -2, -1, 0, 1, 2, 3)

Type	No.	Item	Scoring anchors
Spatial presence	2	Somehow, I felt that the virtual world surrounded me.	Completely disagree → completely agree
Spatial presence	3	I felt that I was only perceiving images.	Completely disagree → completely agree
Spatial presence	4	I did not feel present in the virtual space.	Did not feel present → felt present
Spatial presence	5	I had a sense of acting in the virtual space, rather than operating something from outside.	Completely disagree → completely agree
Spatial presence	6	I felt that I was in the virtual space.	Completely disagree → completely agree
Involvement	7	While roaming in the virtual world, to what extent could I still perceive the external real world, such as sounds, room temperature, or other people?	Very aware → completely unaware
Involvement	8	I was not aware of my real environment.	Completely disagree → completely agree
Involvement	9	I still paid attention to the real environment.	Completely disagree → completely agree
Involvement	10	I was completely absorbed by the virtual world.	Completely disagree → completely agree
Experienced realism	12	To what extent was the experience in the virtual environment consistent with the experience in the real world?	Inconsistent → very consistent
Experienced realism	13	How real did the virtual world seem to you?	As real as an imagined world → indistinguishable from the real world
Experienced realism	14	The virtual world seemed more real than the real world.	Completely disagree → completely agree

Note: The questionnaires listed in this appendix were used during the system development and pre-evaluation stage to help optimize the virtual agent's response quality, usability, and immersive experience design. These questionnaires were not prespecified outcome measures of this randomized trial, nor were they statistically analyzed as formal outcome variables among the 60 randomized participants. Therefore, these materials are provided only as supplementary information for system development transparency and are not used as empirical evidence for the intervention effects or mechanisms of action in this study.
